# Ergogenic effects of quercetin supplementation in trained rats

**DOI:** 10.1186/1550-2783-10-3

**Published:** 2013-01-14

**Authors:** Rafael A Casuso, Antonio Martínez-Amat, Emilio J Martínez-López, Daniel Camiletti-Moirón, Jesus M Porres, Pilar Aranda

**Affiliations:** 1 Department of Health Sciences, University of Jaén, Jaén, E-23071, Spain; 2 Department of Physiology, School of Pharmacy and Institute of Nutrition and Food Technology, University of Granada, Campus Universitario de Cartuja s/n, Granada, 18071, Spain; 3 Department of Music, Plastical Expression and Body Language, University of Jaén, Jaén, E-23071, Spain

## Abstract

**Background:**

Quercetin is a natural polyphenolic compound currently under study for its ergogenic capacity to improve mitochondrial biogenesis. Sedentary mice have exhibited increased endurance performance, but results are contradictory in human models.

**Methods:**

We examined the effects of six weeks of endurance training and quercetin supplementation on markers of endurance performance and training in a rodent model. Rats were randomly assigned to one of the following groups: placebo+sedentary (PS), quercetin+sedentary (QS), placebo+endurance training (PT) and quercetin+endurance training (QT). Quercetin was administered at a dose of 25 mg/kg on alternate days. During six weeks of treatment volume parameters of training were recorded, and after six weeks all groups performed a maximal graded VO_2_ max test and a low-intensity endurance run-to-fatigue test.

**Results:**

No effects were found in VO_2_ peak (p>0.999), nor in distance run during low-intensity test, although it was 14% greater in QT when compared with PT (P = 0.097). Post-exercise blood lactate was increased in QT when compared with PT (p=0.023) and also in QS compared with PS (p=0.024).

**Conclusions:**

This study showed no effects in VO_2_ peak, speed at VO_2_ peak or endurance time to exhaustion after six weeks of quercetin supplementation compared with placebo in trained rats. Quercetin was show to increase blood lactate production after high-intensity exercise.

## Background

Flavonoids are a large family of phenolic compounds or polyphenols with wide therapeutic applications [1]. Quercetin is one of the most widely spread naturally occurring flavonoids, found in onions, garlic, cabbage, leek, broccoli, apples, blueberries, tea and red wine [2]. It is known that quercetin may exhibit anti-oxidant properties due to its chemical structure, particularly the presence and location of the hydroxyl (-OH) substitutions [3]. Despite the fact that after long-term intake there is a wide distribution of quercetin (including its metabolites) in all tissues [4], toxic effects have not been reported until the dose reached 157 mg per kg/d [5].

Quercetin might improve endurance performance since it is known that some polyphenols like quercetin [6] and resveratrol [7] improve aerobic capacity of skeletal muscle by promoting mitochondrial biogenesis in mice. A psychostimulant effect of quercetin has also been reported in vitro [8] in a manner similar to that of caffeine [9], but this effect was not found in human subjects [10]. Quercetin has also been shown to reduce illness after strenuous exercise [11], as corroborated by Davis et al. [12] in a mice model. However, these anti-inflamatory effects seen in vivo are not as powerful as those previously described in vitro [13]. The differences are even greater when the in vivo data is obtained from athletes [14-16].

Quercetin supplementation improves running time to fatigue by stimulating mitochondrial biogenesis in mice [6]. However, this effect has not been observed in humans [16-18]. Research has shown improvements of 3.9% in VO_2_ peak and 13.2% in time to fatigue [19], as well as 2.9% in a maximal 12-minute test after an hour of preload [18] in untrained subjects. These findings are in contrast to those of previous studies [11,17,20]. When athletes are studied, most research has failed to find an ergogenic effect [15,16], in contrast to that of a study of elite cyclists, who exhibited an improvement of their aerobic performance [21]. Finally, effects of quercetin on pre-exercise and post-exercise blood lactate have not been reported [22].

Based on the data provided, the question arises: could quercetin be an ergogenic supplement for athletes or untrained subjects? Our primary goal is to study, for the first time and using a rat model, the effects of both endurance training and chronic quercetin supplementation on 1) endurance capacity, VO_2_ peak, and lactate production, 2) endurance training progress, and 3) distance covered in a low-intensity treadmill test and in a high-intensity treadmill test.

## Methods

### Animals and experimental design

Thirty-three young (three week old) male Wistar rats were randomly allocated into four groups: quercetin and endurance training (QT, n=9), placebo and endurance training (PT, n=8), quercetin and sedentary (QS, n=8), and placebo and sedentary (PS, n=8). Animals, with an initial body weight of 150 (SD=10) g, were housed in individual stainless steel metabolism cages. The cages were located in a well-ventilated thermostatically controlled room (21 ± 2°C), with relative humidity ranging from 40 to 60%. A reverse 12 h light-12 h dark cycle (08.00-20.00 hours) was implemented to allow exercise training during the day. Throughout the experimental period, all rats consumed water and food ad libitum. Two weeks before the experimental period, rats were allowed to adapt to the diet and experimental conditions, and a week before the experimental period, rats had three days of acclimation to the treadmill. Body weight was measured twice per week during this time. After six weeks of treatment we performed two different exercise tests. Tests were carried out after the treatment so that we could compare four different conditions without assessing the effect of training. The reason for choosing a rat model is that a previous study showed that sedentary mice exhibited higher endurance performance with quercetin intake than with placebo [6]. All experiments were undertaken according to the Directional Guides Related to Animal Housing and Care (European Community Council, 1986), and all procedures were approved by the Animal Experimentation Ethics Committee of the University of Jaén.

### Quercetin treatment

Rats were supplemented, during the training period, with quercetin (QU995; Quercegen Pharma, Newton, MA, USA) on alternate days at a dose of 25 mg/kg. This dose has been reported to improve mitochondrial biogenesis and endurance capacity in sedentary mice [6]. Quercetin was diluted in a 1% solution of methilcellulose, and was administered using a metal gavage. Oral gavage was performed to ensure that 25 mg/kg of quercetin was introduced into the stomach. Quercetin also contained vitamins B3 and C, which have been shown to increase the bioavailability of quercetin (personal communication, Quercegen Pharma). The PT and PS groups were also supplemented with methilcellulose and vitamin B3 and C with the same concentration as in QT and QS.

### Training protocol

Trained animals were exercised five days per week during six weeks on a motorized treadmill (Panlab TREADMILLS for five rats LE 8710R). We followed a modification of the protocol of Davies et al [23]. Animals ran at a constant speed of 44 cm/s and at 10% grade. The first day's training session was 20-minutes long, and every two days the work period was increased by five minutes. On the last day of the fifth week they were required to run for a full 80 minutes. This work duration was maintained during the sixth week. The untrained group was exercised at the same speed and grade for only 10 minutes twice per week, in order to ensure that they were able to perform the tests performed at the end of the treatment.

Twenty-four hours after the last training session, all animals performed a graded high-intensity treadmill test to determine VO_2_ peak using a treadmill gas analyzer (Model LE405, Panlab/Harvard Apparatus) previously calibrated with mixtures of O2 and CO2 at different concentrations. After an initial two minutes with no grade at 22 cm/s, treadmill speed was increased by 11 cm/s every two minutes. The test was finished when the rat was exhausted and located at the end of the treadmill, on the shock bar, for 5 seconds, when rats were quickly removed [24]. VO_2_ peak was defined as the highest 20” interval recorded during the test. Blood lactate was measured before and immediately after the test using a Lactate-Pro analyzer, blood was taken from a small cut in the rat's tail.

After twenty-four hours of recovery a low-intensity endurance test was performed. Each rat was required to run to exhaustion at 44 cm/s at a 10% grade. The test finished when the animal was visibly exhausted, not able to maintain the appropriate pace, and this resulted in a rising frequency of landings on the electrical shock grid [24]. The endpoint was marked by the rat's inability to return to the treadmill belt, and to stand on a flat surface.

### Statistical methods

Treatment effect between trained (QT vs PT) and sedentary (QS vs PS) groups was analyzed with a t test for independent samples, using study groups as independent variables and each of the performance parameters measured as dependent variables (Weight, VO_2_ peak, vVO_2_ peak, maximum speed achieved, time of endurance test, distance run and distance run until RQ= 1, and VO_2_ at exhaustion). Lactate production measured before and after the maximal incremental treadmill test was analyzed using a two-way repeated measures ANOVA, with groups as between-subject variable and exercise time as within-subject variable. When the effect was significant, post hoc analysis was performed and adjustment done through the Bonferroni confidence interval. The level of significance was P≤0.05 for the t-test and P≤0.008 in post hoc Bonferroni's comparisons (P=0.008 needed for significance with an experiment-wise alpha of 0.05 using Bonferroni adjustment in alpha for six comparisons). All analyses were performed using the Statistical Package for Social Sciences (SPSS, version 19.0 for Windows; SPSS, Inc., Chicago, IL, USA).

## Results

### Training progress

**Figure 1:**
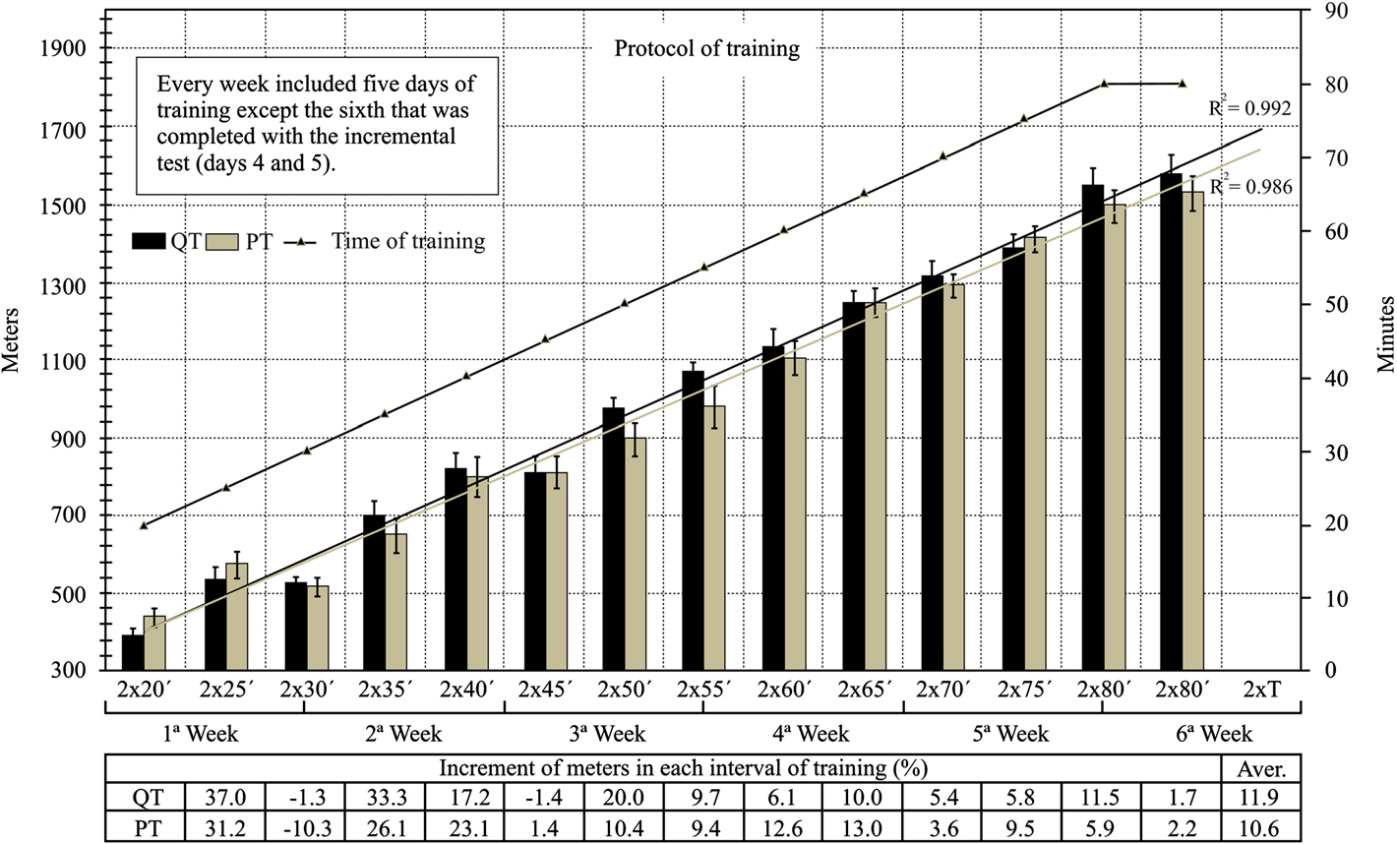
**Training protocol of six weeks for rats.** No significant difference (P>0.05) in distance run between QT and PT at any stage of training. ‘ = Minutes, Aver = Average, T= Application of tests. The percentage of increase in distance run was computed as ((interval - previous interval) / previous interval) x 100.

The training protocol and the effect of time on the meters run is presented in Figure 1. The QT and PT groups were subjected to a six-week duration training with an increase of five minutes every two days up to a maximum of 80 minutes, which represented an average increase of the load between intervals of 11.9 and 10.6% in QT and PT respectively. The final training volume increased by 399% to 349% in QT and PT compared with baseline. There were no differences in the distance run by the two groups at any time of training (P> 0.05). The average/day of meters walked were 986 and 1002 in the QT and PT groups respectively. Although the relationship between training time and distance covered showed an almost linear fit in both groups (R2 = 0.992 and 0.986) for QT and PT respectively, there was a sligh improvement in the performance of the QT group.

### Endurance capacity

**Figure 2:**
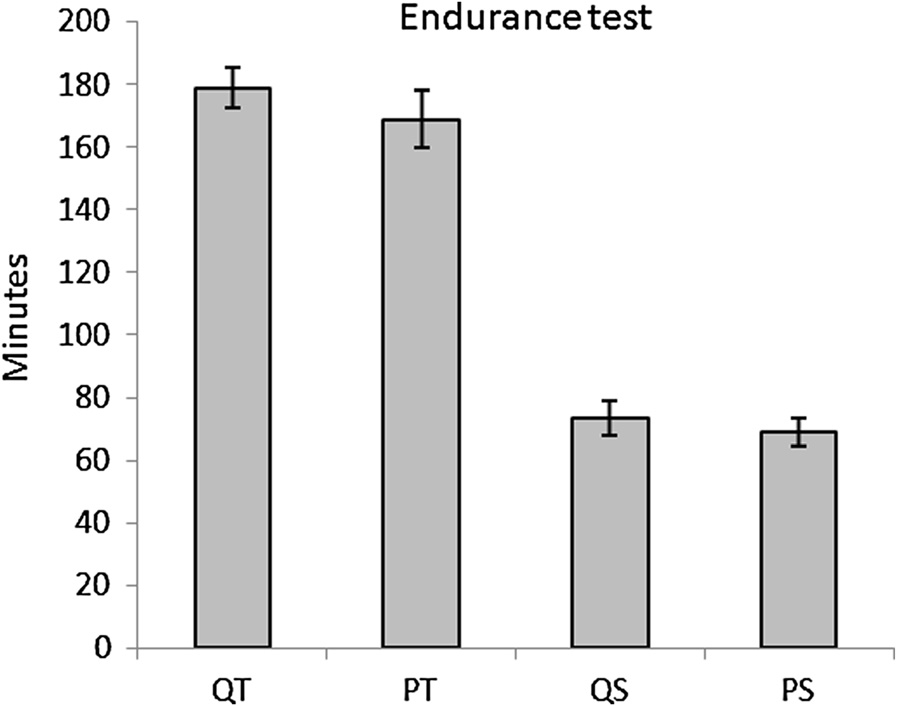
**Time run until exhaustion in the low-intensity endurance regime.** T- test for independent samples reported no significant differences between QT and PT or QS and PS (P>0.05).

**Figure 3:**
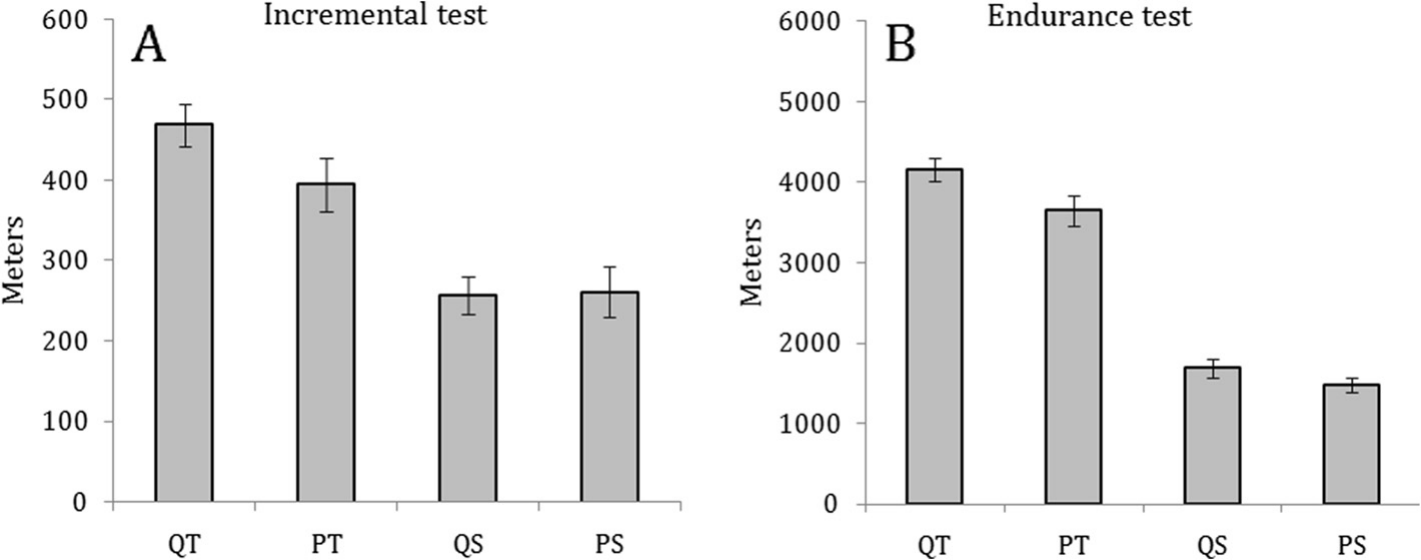
**Distance run until exhaustion in A) high-intensity incremental test and B) low-intensity endurance test. ** T-test for independent samples reported no significant differences between QT and PT or QS and PS (P>0.05).

There were no significant difference in exercise performance between the quercetin and placebo trials. Although the QT group ran for 5.91% longer (Figure 2) and 14% further (Figure 3B) than the PT group, there were no significant differences in either time [P=0.351, Power=0.147] or distance [P=0.051, Power=0.512)].

### Maximal incremental test

During the incremental test VO_2_ peak, speed at VO_2_ peak and maximum speed achieved did not differ between quercetin and placebo conditions (Table 1). There were no differences between the final weight after treatment, as shown in Table 1. Although the distance achieved by QT was 18.6% greater than PT this result was not significant [P=0.102, Power=0.380] (Figure 3A).

**Table 1:** Mean value (standard deviation) after incremental maximal test

	**Trained**						**Sedentary**					
	**QT**	**PT**	**t**	**df**	**P**	**Power**	**QS**	**PS**	**t**	**df**	**P**	**Power**
**WEIGHT (g)**	352.89±31.25	367.25±24.41	1.045	15	0.312	0.161	379.25±52.91	366.63±8.97	0.595	7.298	0.570	0.086
**VO**_**2**_**MAX (ml/kg/min)**	63.55±8.58	58.62±7.38	1.272	14.990	0.223	0.219	65.12±8.21	61.87±5.51	0.929	14	0.369	0.139
**/vVO**_**2**_**MAX (cm/s)**	47.89±8.17	48.50±16.18	0.100	15	0.922	0.051	46.88±13.21	46.63±10.98	0.041	14	0.968	0.052
**MAX. VEL (cm/s)**	95.11±7.40	87.50±9.65	0.837	15	0.086	0.405	71.63±8.68	71.63±11.01	0.002	14	0.998	0.050

Compared values for trained (QT vs PT) and sedentary groups (QS vs PS). T-test for independent samples reported no significant differences between QT and PT or QS and PS. VO_2_ MAX: Maximum oxygen uptake; vVO_2_ MAX: Velocity at VO_2_ max; MAX.VEL: Maximal velocity achieved. df: degrees of freedom. Power: statistical power.

**Figure 4:**
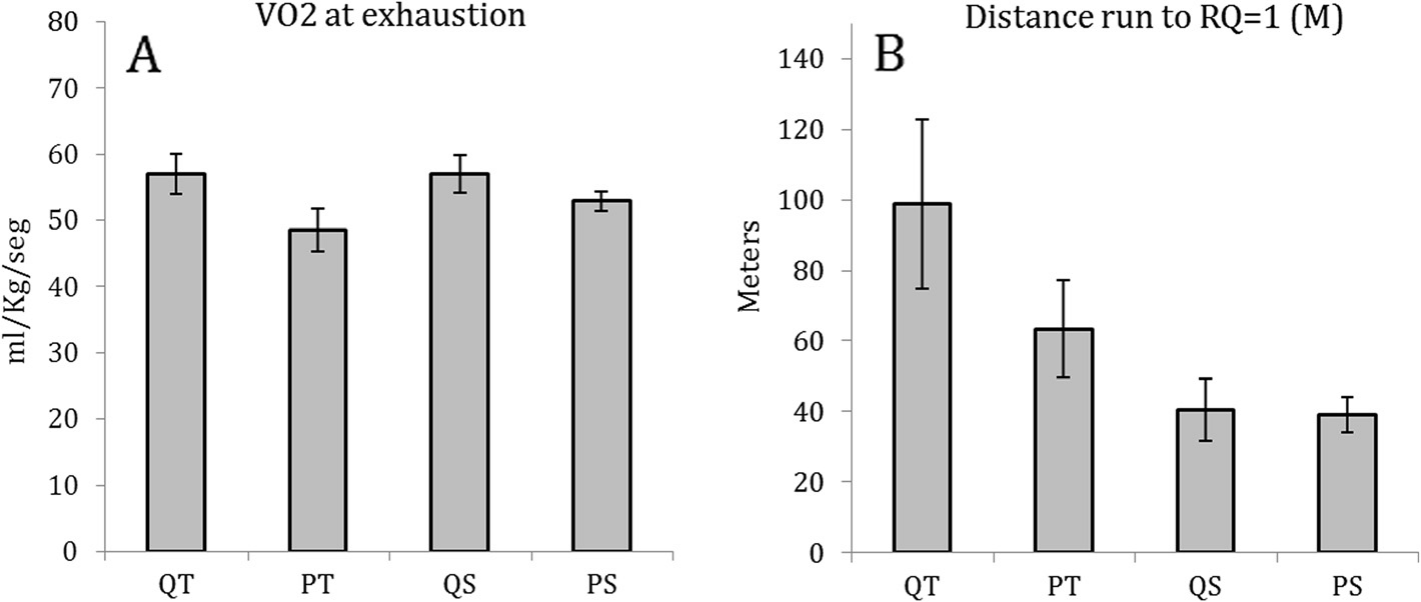
**A) VO**_**2 **_**at the end of the high-intensity incremental test B) Distance run until RQ=1.** T-test for independent samples reported no significant differences between QT and PT or QS and PS (P>0.05).

**Figure 5:**
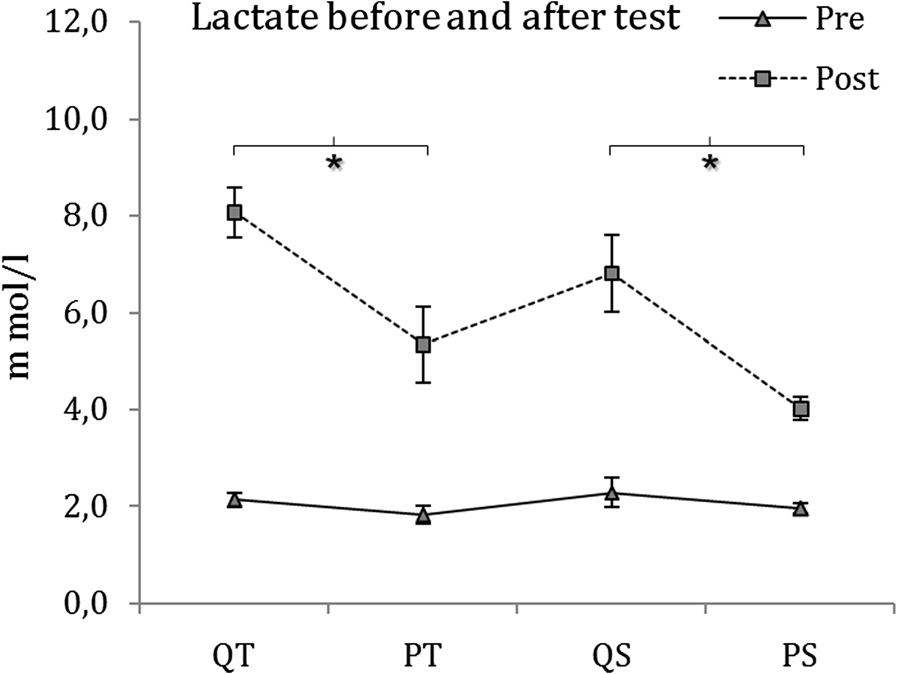
**Blood lactate pre- and post-exercise using a two-way repeated measures ANOVA.** (P=0.008 needed for significance with an experiment-wise alpha of 0.05 using Bonferroni adjustment in alpha for six comparisons) * Post lactate differences (P=0.004) in QT vs PT and QS vs PS.

Figure 4B shows that the QT group ran for 56.1% longer before reaching RQ=1 compared with the PT group, but this effect was not significant [P=0.222, Power=0.213]. Similar results are illustrated by Figure 4A, in which VO_2_ at exhaustion does not differ after the high-intensity test for the quercetin and placebo exercise groups (P=0.069, Power=0.448). Lactate production was analyzed (pre- and post-high-intensity test) using repeated measures ANOVA, where we observed a group effect P=0.001, Power=0.967 and a group interaction per time unit P=0.001, Power=0.977. Specifically, lactate production immediately after the high-intensity test was increased in the QT and QS groups compared with the PT and PS groups (P=0.004) [Figure 5]. No differences were found in lactate production between groups prior to the high-intensity test (P>0.05). Lactate production was significantly increased in each group (P<0.001 in QT, QS y PS) and (P=0.004 in either PT) at the end of the high-intensity test (data not shown).

## Discussion

A recent study evaluated the effects of short-term quercetin supplementation on exercise performance in mice [6] and demonstrated a significant increase in endurance capacity and mitochondrial biogenesis in comparison with placebo groups. Using a rat model, no significant performance effect (VO2 peak, endurance capacity and training parameters) was measured in trained rats with a quercetin dose of 25 mg/kg on alternative days compared with placebo.

The endurance training protocol used in this study was a modification of a widely used protocol in the literature [23,25,26]. As shown in Figure 1, distance run increased with time. These data suggest that the training workload was well adjusted, since a plateau in the training volume is a sign of overtraining [27]. No difference was found in the average daily distance run between the QT and PT groups. VO_2_ peak values in rats vary depending on the methodological test used or on their weight [28]. Our results show that six weeks of quercetin supplementation did not increase VO_2_ peak or VO_2_ at exhaustion in sedentary or trained rats. It must be noted that our protocol did no alter inclination in order to examine the maximum speed achieved. Protocols that do not use an incline are known to induce a lower VO_2_ peak than others with 15°-20° inclination [28,29]. However, our results were similar to those recently reported [17], but were in contrast with the ones that reported an increase of VO_2_ peak by quercetin in sedentary humans [19]. Speed at VO_2_ peak was also analyzed in this experiment, with no change reported in the quercetin groups. We hypothesized that quercetin would increase VO_2_ peak due to its ability to increase mitochondrial biogenesis in mice (6). However, as described above, no differences were observed in any groups on measures related to oxygen uptake by quercetin supplementation. These results are similar to those obtained by Bigelman et al [30]. There are several potential reasons for these results: firstly, VO_2_ peak is influenced by muscle mitochondrial oxidative capacity, but relative to endurance capacity, it is limited to a greater extent by oxygen delivery via the cardiovascular system [31]. Secondly, larger doses over extended periods using added flavonoids such as eppigallocatechin gallate (EGCG) may augment quercetin's effects on mitochondrial biogenesis. This could be a more appropriate supplement to increase oxygen consumption [16]. However, previous work did not find any ergogenic effect of quercetin and EGCG supplementation in a moderately trained sample [30].

To examine additional ergogenic effects of quercetin in rats, oxygen consumption and carbon dioxide production were measured during the incremental exercise test. This enabled the calculation of RQ. In all groups of rats, the average RQ remained fairly constant and did not differ between groups (data not shown). When VCO_2_ is greater than VO_2_ (RQ>1.0), this point of inflection is correlated with blood lactate accumulation [32]. QT group showed a trend to run longer before reaching an RQ of 1.0 (Figure 4B) indicating that these rats were able to use oxidative metabolism for a longer period.

Fatigue in the endurance test is thought to arise primarily from limitations in the periphery, like the cardiovascular system and muscles [6]. Although it has been reported that antioxidant supplementation may decrease endurance performance [25], the trained groups showed an increase in time to fatigue of 244.96% and 244.93% for QT and PT respectively when compared to PS. However, in contrast with others [6], we did not observe an improvement in QS. When compared to trained groups, there was a non-significant increase of 5.91% in the QT group in time to fatigue. Despite being non-significant, this result was related to recently published results by Kesser et al. [33].

We employed two different types of exercise (a low intensity endurance capacity test and a maximal graded intensity test). Although both are commonly used exercise models, the stimuli are totally different. During the treadmill running endurance test mice run at a given intensity until they can no longer maintain the pace and end up on the electrical shock grid [24,25]. The performance in this type of exercise is known to be related to the oxidative capacity of muscles. However, during the maximal progressive intensity test, rats achieved higher velocities, a performance reflecting their capacity to use glycogen as a source of fuel. Distance run to exhaustion was recorded during these two different regimes (Figure 3). Under the high-intensity regime (test used to analyze oxygen consumption) the QT group ran (18,6%) longer than PT. Under the low-intensity regime (endurance test) QT ran 14% (p=0.097) further than PT. These results were not significant, however they demonstrated a trend that may become significant after a longer treatment.

Although no effects have been previously reported [22], the present study demonstrated that quercetin had an effect on blood lactate immediately after exhaustion. When the QT and QS groups reached exhaustion, their blood lactate levels were elevated when compared with PT and with PS respectively (Figure 5). These elevated blood lactate levels were an indication of enhanced glycolysis and lactate production in the skeletal muscle [30] in the quercetin supplemented groups that had run to exhaustion. However, there are other possible reasons that may explain the quercetin effects in addition to improvements in glycolytic flux. The psychostimulant effects of quercetin [8] could increase effort at high intensities and this could result in an increased lactate production. However, further experiments may corroborate this quercetin effect by measuring glycogen depletion in muscle and liver during high-intensity exercise.

In summary, no effects were measured in VO_2_ peak, speed at VO_2_ peak or endurance time to exhaustion after six weeks of quercetin supplementation compared with placebo in trained rats. No effects were found either in sedentary rats supplemented with quercetin compared with placebo. However, a trend was visible regarding increased performance by quercetin supplementation in some parameters like distance run until exhaustion or distance run until RQ=1. Perhaps after longer treatment, like eight or ten weeks, this effect could be significant. For the first time we have detected an increase in blood lactate production by quercetin, although more research is needed on this topic. No effects on exercise performance were found but this will need to be verified by further studies examining muscle physiology.

### Limitations and strengths

The present study has several limitations that must be mentioned. First, the present physiological results obtained in rats must be confirmed in human subjects after long-term quercetin ingestion, since our results cannot be extrapolated to the potential effects over months in trained human subjects. Also, there is a lack of evidence regarding how much quercetin must be supplemented for it to exert its ergogenic effects, although 25 mg/kg is thought to be a good start. In addition, the six-week protocol applied may be insufficient to observe any ergogenic effect, and in fact there are some parameters that started exhibiting a trend and might be significant after 8-13 weeks of treatment. Finally, the lower statistical power observed in most of our results suggests to be cautious in interpreting them, future research with larger samples are needed to draw definitive conclusions. On the other hand, this is the first research that has analyzed the effect of quercetin on both sedentary and trained rats, hopefully paving the road for studies intended to find out if quercetin supplementation can enhance performance in trained athletes.

## Competing interests

The authors declare no competing interest.

## Authors’ contributions

RAC was involved in the conception, design, acquisition and analysis of the data and drafting the manuscript, AM-A was involved in the conception, design, acquisition and analysis of the data and drafting the manuscript, EJM was involved in the conception, design, acquisition and analysis of the data and drafting the manuscript, DC-M was involved in the conception, design, acquisition and analysis of the data and drafting the manuscript, JMP was involved in the analysis of the data and drafting the manuscript and PA was involved in the conception, design, acquisition and analysis of the data and drafting the manuscript. All authors have given final approval of the version to be published.
